# Mutational Analysis of a Familial Adenomatous Polyposis Pedigree with Bile Duct Polyp Phenotype

**DOI:** 10.1155/2021/6610434

**Published:** 2021-04-12

**Authors:** Li-jun Xie, Dan-dan Ruan, Jian-hui Zhang, Yi Li, Li Chen, Mao-lin Yan, Ming-dian Yu, Jie-wei Luo, Hui-zhen Zhang

**Affiliations:** ^1^Department of Oncology of Zhangzhou Traditional Chinese Medicine Hospital, Zhangzhou 363000, China; ^2^Shengli Clinical Medical College of Fujian Medical University, Fuzhou 350001, China; ^3^Fujian Provincial Hospital, Fuzhou 350001, China; ^4^Department of Pharmaceutics, School of Pharmacy, China Pharmaceutical University, Nanjing 210009, China; ^5^Department of Ultrasound, South Hospital of Fujian Provincial Hospital, Fuzhou 350001, China

## Abstract

A large number of colorectal cancers have a genetic background in China. However, due to insufficient awareness, the diagnostic rate remains low and merely 5-6% of colorectal cancer patients are diagnosed with hereditary colorectal cancer. Familial adenomatous polyposis (FAP) is an autosomal dominant genetic disease caused by mutations in the adenomatous polyposis coli (APC) gene. Different mutation sites in APC are associated with the severity of FAP, risks of carcinogenesis, and extraintestinal manifestations. We used next-generation sequencing (NGS) and capture techniques to screen suspected mutation points in the proband in this pedigree. Using modified Sanger sequencing, we identified members of the family who were carriers of this variant and whether this segregated well with disease occurrence. FAP family members had multiple adenomatous polyps in their gastrointestinal tracts, some of which developed into cancer with age. Two subjects presented a rare common bile duct polyp phenotype. No extraintestinal manifestations were observed. A heterozygous frameshift mutation in APC exon 16 (NM_000038.6) was observed in the proband and in other patients: c.3260_3261del (p.Leu1087GlnQfs^*∗*^31) (rs587782305); the variant call format was CCT/C. Due to the deletion of two bases, a stop codon appeared after 31 amino acids, and the protein was truncated prematurely, which affected the conformation of the protein. Pedigree genetic linkage analysis showed that the clinical phenotype cosegregated with the APC mutation p.L1087fs. This mutation may be the pathogenic in this FAP family and responsible for this rare common bile duct polyp.

## 1. Introduction

In China, the morbidity and mortality rates of colorectal cancer have been on a rise; many of these cases have a genetic background. However, only 5-6% of patients are diagnosed with hereditary colorectal cancer in China [1]. Due to the inadequate awareness regarding this disease in China, the actual rate of hereditary colorectal cancer remains low. Hereditary colorectal cancer characterized by polyposis includes familial adenomatous polyposis (FAP), Peutz–Jeghers syndrome (PJS), juvenile polyposis syndrome (JPS), and serrated polyposis syndrome (SPS); it can be further divided into classical FAP (CFAP), attenuated FAP (AFAP), MUTYH-associated polyposis (MAP), Gardner syndrome, and Turcot syndrome subtypes. Clinically, FAP (including CFAP and AFAP) is the most commonly observed syndrome [2].

FAP was first reported in 1925 [3]. It is a special type of hereditary colorectal cancer characterized by a highly explicit autosomal dominant inheritance. FAP is characterized by an early onset, and it can manifest even in newborns, with no apparent gender orientation. FAP manifests as a diffuse growth of hundreds or thousands of adenomatous polyps in the colorectal mucosa, without observable early symptoms. However, with the passage of time, the number and size of polyps continue to increase, along with the emergence of abdominal symptoms such as pain, diarrhea, and obstruction in the intestine. More than 70% patients may have extraintestinal manifestations of congenital hypertrophy of the retinal pigment epithelium (CHRPE), multiple osteoma, and dental deformity. In the past, FAP has also been termed Gardner syndrome, Turcot syndrome, gastric adenocarcinoma, or proximal polyposis of the stomach. If left untreated, the classic FAP malignancy can reach as high as 100% around the age of 40 [4, 5], and the average colorectal carcinoma (CRC) age of onset of AFAP with slow polyps development is approximately 55 years [6]. The risk of cancer increases by 2.4-fold every 10 years, and the optimal age for surgery is before 25 years of age; hence, early detection is of great significance [7]. Prevention, early diagnosis, and early treatment can be achieved through the detection, genetic diagnosis, and risk management of the probands of families as well as through genetic screening and follow-up monitoring for other family members.

## 2. Materials and Methods

### 2.1. Research Subjects

**Figure 1:**
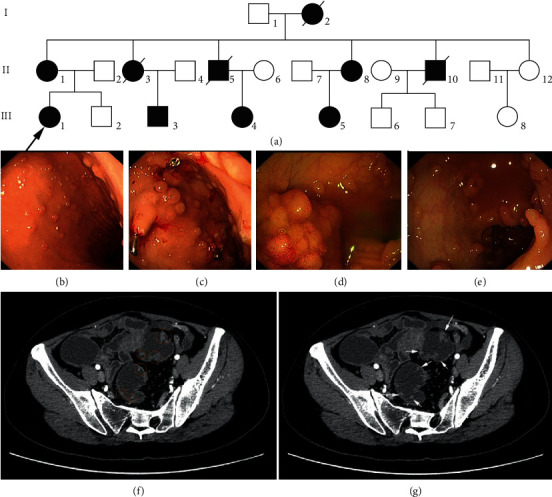
(a) Familial adenomatous polyposis (FAP) family pedigree. Proband (III1) had multiple sites of adenomatous polyposis of the colon and rectum at 28 years of age, (b), (c) gastroscopy and (d), (e) colonoscopy, was diagnosed with “colon cancer” at age 31. Cohort members I2, II3, 5, and 10 all died of “colon cancer and adenomatous colonic polyposis.” Members II8, III4, 5, and 3 had adenomatous colonic polyposis. Members I2 and II8 also suffered from adenomas of the common bile duct. (f), (g) CT images of II8 before total colorectal resection; the images show changes in the distal stomach, pancreas, and duodenum, polyps in the rectum and sigmoid colon wall, and the heterogeneous fatty liver.

In this study, we investigated an FAP family pedigree (Figure 1(a)). The proband (III1) visited our hospital 6 years ago, and we then conducted a genealogical investigation. Endoscopy of the proband (III1) at 28 years of age revealed several hundred polyps in the colon and rectum and was considered for FAP diagnosis (Figures 1(b)–1(e)). At age 31, colon cancer was identified, and total colectomy + ileorectal anastomosis was performed. Pedigree analysis revealed a family history of familial gastrointestinal polyposis and colon cancer. The grandmother (I2) of the proband had a history of “bile duct polyps and adenomatous polyps of the colon” and died of “colon cancer.” Five out of six siblings in the second generation also had the illness; three of them (II3, 5, and 10) died of “colon cancer and adenomatous polyps of the colon.” The mother (II1) had multiple adenomatous polyps in her gastrointestinal tract at the age of 32 years and underwent total colectomy and ileorectal anastomosis when colon cancer was diagnosed at an age of 52 years. Endoscopy results for one of the aunts (II12) suggested an ectopic gastric mucosa in the upper esophagus. The female cousins (III4 and 5, aged 24 and 25 years old) and male cousins (III3, 40 years old) had colon adenomatous polyps. Another aunt (II 8) of the proband had undergone “laparoscopic cholecystectomy + common bile duct exploration + common bile duct mass resection + T-tube drainage” in November 2012 at the age of 40 years because of “right upper abdominal pain, acute cholangitis, and space-occupying lesion in the common bile duct.” Multiple polypoid lesions were observed in the lower part of the common bile duct during the operation. Postoperative pathology showed that adenoma in the lower segment of the common bile duct was accompanied by mild to moderate atypical hyperplasia of the glandular epithelium. Three months later, the common bile duct adenoma was resected through the T-tube sinus tract. Postoperative pathology showed villous adenoma of the bile duct with moderate to severe atypical hyperplasia. In July 2013 (at age 41), the aunt was admitted to Fujian Provincial Hospital with the main complaint of “repeated abdominal pain for two years plus fever for last three days.” Her test results showed normal serum bilirubin, alanine aminotransferase 168 U/L, aspartate aminotransferase 88 U/L, alkaline phosphatase 858 U/L, and glutamyl aminotransferase 520 U/L. Magnetic resonance cholangiopancreatography (MRCP) showed irregular intrahepatic and extrahepatic bile duct dilatation, with the greatest common bile duct diameter of 1.7 cm. In addition, multiple space-occupying lesions of the common bile duct were observed. A diagnosis of “acute cholangitis, common bile duct villous adenoma, and possible FAP” was made, and pancreatoduodenectomy was performed; this was published by us as a case report [8]. In 2014 (aged 42), she was admitted again due to “increased number of bowel movements with bloody stools for more than 10 years” and a diagnosis of “multiple gastrointestinal polyps: FAP possible.” Total colorectal resection was performed (Figures 1(f) and 1(g)). With the approval of the Fujian Provincial Hospital Ethics Committee (K2015-031-01), each member of the FAP family undergoing investigation signed an informed consent form.

### 2.2. Clinical Phenotype Detection

We collated clinical manifestations and related biochemical tests of the proband and family members, including results of ultrasound, computed tomography (CT), nuclear magnetic resonance imaging (MRI), and gastrointestinal endoscopy.

Histology and immunohistochemistry: the collected tissues were dehydrated routinely, embedded with paraffin tissue blocks, and sliced continuously. The slices with thickness of 3-4 *μ*m were stained with hematoxylin-eosin staining (H&E), and the slices with the thickness of 2-3 *μ*m were removed on the antistripping slices with positive control and were baked in an oven at 60–70°C for 1-2 h. Dyeing was according to the preset procedure, and colorectal cancer specimens were stained with CK20, CK7, CEA, CDX2, P53, Ki67, and *β*-catenin. The stained sections were routinely dehydrated, transparent, and sealed after cleaning. The automatic immunohistochemistry instrument is Ultra (Roche Company, US). The abovementioned staining protein primary antibodies were purchased from MXB Biotechnology (Fuzhou, China), and the secondary antibodies, chromogenic system, and H&E restaining solution were all equipped with the corresponding instruments.

### 2.3. DNA Extraction

A sample of 12 mL of peripheral blood was collected into EDTA anticoagulant tubes from the proband and other family members who agreed to be investigated. Genomic DNA was extracted according to the instructions of the TIANGEN extraction kit.

### 2.4. Target Region Capture Sequencing and Bioinformatics Analysis

First, the concentration of DNA samples was determined using Nanodrop 2000, and DNA fragmentation was performed. Next-generation sequencing (NGS) and sequence capture technology were used to detect the proband. TargetSeq® liquid-phase chip capture sequencing is a target region gene detection project developed by iGeneTech®. TargetSeq® designs the target region genome based on a multifactor algorithm and then synthesizes effective specific probes, which are hybridized with genomic DNA in the liquid phase. After the target region sequence is captured and enriched, mainstream sequencing platforms such as Illumina are used for high-throughput sequencing. By sequencing the target region, candidate genes or candidate sites can be detected. The DNA fragments were sheared and recovered using Covaris, and an Illumina sequencing library was constructed. DNA capture microarray containing multiple genes underwent multiple rounds of targeted gene enrichment followed by DNA sequencing (Illumina MiSeq). Short oligonucleotide analysis package (Soap) software was used to analyze the copy number, polymorphisms, and insertion/deletion data to screen for suspected disease-causing mutations. SIFT (http://sift.jcvi.org/) and Polyphen software (http://genetics.bwh.harvard.edu/pph/) were used to predict the effect on the function of mutant proteins. The above steps were completed in collaboration with Beijing Bestnovo Medical Technology Co. Target genes included APC, EPCAM, and MUTYH, and the related genetic and colorectal cancer pathogenic gene coding regions and flanking regions were detected (T192V1Plus, liquid phase analysis platform, iGeneTech, Beijing, China).

### 2.5. Sanger Sequencing Validation

Polymerase chain reaction (PCR) was performed to amplify the fragments of suspected candidate mutation loci, and Sanger DNA sequencing validation was performed to detect the corresponding loci in the proband and family members participating in the study. Primer Premier 5 software was used to design the target sequence primers. The APC sequence was obtained from GenBank (NM_000038.6), and the target amplicon length was 446 bp in APC exon 16 (c.3260_3261del: p.L1087fs). The following primers were used: F: 5′-TCAGATGAGCAGTTGAACTCTGGAAGG-3′ and R: 5′-CTATAATCAATAGGCTGATCCACATGAC-3′. The PCR was performed in a 50 µL reaction volume to amplify the target fragments on a thermocycler instrument (PTC-200 PCR, BioRad), and the annealing temperature of PCR was 58°C. The PCR products were purified using Takara reagents, and the PCR products of the target fragments were sequenced on an ABI 3730XL platform. The DNA extracted from the pedigree members (II1, II2, II8, II12, III1, III3, III4, III5, III6, III7, and III8) was subjected to PCR amplification and Sanger sequencing in the target region to detect whether they carried the frameshift mutation p.L1087fs.

## 3. Results

### 3.1. FAP Family Pedigree Analysis

**Figure 2:**
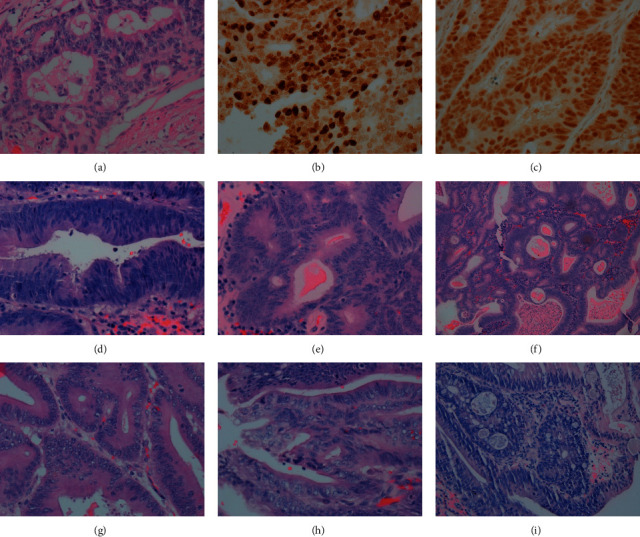
Pathology image. III1, postoperative pathology of colon cancer showed raised tubular adenocarcinoma grade II. (a) H&E staining, × 400, Ki67 (60%+, × 400), (b) P53 (wild type, × 400), (c) II8, common bile duct adenoma with high-grade intraepithelial neoplasia (d) × 400, (e) × 400, and (f) × 100, papillary tubular adenoma of duodenal papilla with local high-grade intraepithelial neoplasia (g), (h) × 400, and colon tubular adenoma-like polyp (i) × 400.

At age 28, the proband (III1) was diagnosed to have colon and rectal polyposis, which developed into frank “colon cancer” after three years. Pathology investigations after total colon + partial rectal resection revealed stage II tubular adenocarcinoma of the large intestine, with perineural invasion, vascular cancer thrombi, and invasion of the subserosal layer accompanied by extensive adenoma polyps in the large intestine. Three out of 28 lymph nodes from the large intestine exhibited cancer metastasis. Immunohistochemistry revealed CK20 (+++), CK7 (+), CEA (+++), CDX2 (+++), P53 (wild type), Ki67 (60%+), and *β*-catenin (membrane+). Elastic fiber staining (+) showed that the malignant tissue had infiltrated into the venous duct structures (Figures 2(a)–2(c)). A rare clinical phenotype, adenoma of the common bile duct, was observed in FAP pedigree members I2 and II8. Member II8 underwent biliary tract surgery and coral-like neoplasia was observed in the middle and lower parts of the common bile duct, with a diameter of approximately 1.0 cm, broad-based, which was brittle and easy to bleed. Postoperative pathology showed “villous adenoma of the common bile duct.” Pancreaticoduodenectomy was performed at our hospital. The common bile duct was dilated to a diameter of approximately 2.0 cm, and several hard masses were identified around the duodenal ampulla. Postoperative specimens showed duodenal papilla of approximately 5.0 × 4.0 cm in size, and the texture was firm with discernable borders. Multiple polyps ranging between 0.3 and 0.5 cm in diameter were observed in the descending part of the duodenum. Multiple pedunculated tumors with a diameter ranging from 1.0 to 1.5 cm were found in the common bile duct. Multiple polyps with diameters ranging from 0.5 to 0.7 cm were observed in the gastric wall. Postoperative pathology results showed duodenal papillary tubular adenoma with local high-grade intraepithelial neoplasia and common bile duct tubular adenoma with high-grade intraepithelial neoplasia (Figures 2(d)–2(h)). Colonoscopy indicated multiple colon polyps with a diameter of approximately 0.3–2.5 cm spanning the entire colon, with a greater concentration in the sigmoid colon and rectum. In addition, a sessile flat bulge in the ascending colon was observed, which was considered to be FAP. It had a diameter of approximately 1.5 × 2.0 cm, with nodular surface mucosa. Capsule enteroscopy revealed no abnormalities. Postoperative pathology confirmed FAP of the large intestine. The polyps presented as tubular adenoma with low-grade intraepithelial neoplasia (moderate dysplasia) (Figure 2(i)). All deceased members of the family (I2, II3, II5, and II10) had colon cancer and adenomatous colonic polyposis. Gastrointestinal pathology of member II1 revealed multiple gastrointestinal adenomatous polyps and colonic tubular adenocarcinoma. No extraintestinal manifestations of CHRPE, bone marrow and tooth deformities, epidermoid cysts, lipomas, scleroderma, or other malignant tumors such as thyroid cancer and hepatoblastoma were observed in the family members under investigation.

### 3.2. Gene Analysis of the FAP Family Pedigree

**Figure 3:**
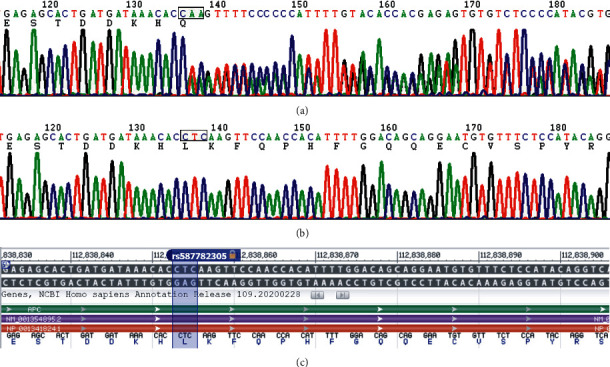
In the FAP family pedigree, a heterozygous deletion was observed in exon 16 of the APC (NM_000038.6): c.3260_3261del: p.(Leu1087Glnfs^*∗*^31). Deletion of two bases (TC) causing a frame shift (a), b is the wild type, and c is the corresponding schematic diagram of genomic regions, transcripts, and products.

Whole exome sequencing of liquid-phase chip capture sequencing was performed on the Illumina platform (target area 42M, covering gene coding region + splicing site + mtDNA gene site). Using Trimmomatic, Bwa, Samblaster, GATK, and other softwares, the splice sequence and low-quality data were removed, and the remaining data were compared with the reference genome. As a result, total of 80.71 M target mapped reads and 13928.71 M total accurate mapped bases were generated. The coverage rate of the target area was 99.76%, and the effective sequencing depth was 168.91. The coverage of 4X, 10X, and 20X was 99.64%, 99.46%, and 99.08%, respectively. We used GATK, SAMtools, VarScan, Annovar, and snpEff to analyze data for SNP, InDel, CNV, and mutation annotation. After selecting known related genes APC, EPCAM, and MUTYH, other gene mutations, and filtering population data and synonymous mutations, the APC mutation was identified. Through NGS detection, the proband revealed a heterozygous frameshift mutation in exon 16 of the APC (NM_000038.6): c.3260_3261del(p.Leu1087Glnfs^*∗*^31) (rs587782305) (Figure 3). The variant call format (VCF) was CCT/C due to the deletion of two bases; a stop codon appeared after 31 amino acids, and the protein was prematurely truncated, which affected protein conformation. It was included in the HGMD database, and its clinical significance was annotated as pathogenic. For more information, refer to http://www.hgmd.cf.ac.uk/ac/gene.php?gene=APC. Mutations are commonly associated with pathogenesis in FAP patients and have been detected in several FAP family pedigrees. Sanger DNA sequencing indicated that patients II1, II8, III1, III3, III4, and III5 carried the frameshifting mutant p.L1087fs, but other family members, II12, II2, III6, III7, and III8 did not. Pedigree gene-phenotype correlation analysis showed that the clinical phenotype cosegregated with the gene mutation p.L1087fs. This supports the classification of this variant as pathogenic.

## 4. Discussion

The earliest identification by Groden confirmed that FAP is directly related to the APC [10, 11]. The APC is located on 5q21-q22 of autosome 5 and contains 16 exons. The 100 kDa APC protein is composed of 2,843 amino acids [12]. Recently, a new type of polyposis syndrome was proposed: gastric adenocarcinoma and proximal polyposis of the stomach (GAPPS). The typical endoscopic manifestation of GAPPS is the presence of polyps at the proximal end of the stomach, whereas the distal end is not affected. Compared with other polyposis diseases, GAPPS can significantly increase the risk of gastric adenocarcinoma, whereas the risk of rectal cancer is low. GAPPS is associated with mutations involving specific sites in exon 1B of the APC [13]. However, incidental extrahepatic adenomatous biliary polyp is very rare in clinical practice, and there are very few published case reports. Except for endoscopic ultrasonography, MRI, and CT, clinical diagnoses at the early stage of the disease is difficult, and the understanding of its natural progress remains limited [14, 15]. In patients with FAP, the incidence rate of adenomas and microadenomas in the duodenal papilla and Vater periampullary region (extending to the extrahepatic bile duct area) is high [16]. The relative risk of duodenal adenocarcinoma and ampullary carcinoma in FAP patients is 331 times and 124 times higher, respectively [17]. Soravia et al. [18] described severe duodenal polyposis in patients with 5′ mutations in the APC. Mutations in the central part of the APC and exon 16 (especially the distal end of codon 1400) make individuals prone to a severe duodenal phenotype [19]. Björk et al. reported 12 APC mutations downstream of codon 1051 in exon 16 in 15 FAP patients, revealing that the mutation downstream of codon 1051 may be related to severe periampullary lesions [20].

APC is a classic tumor suppressor protein. The destructive complex formed by the combination of APC protein with Axin and glycogen synthase kinase-*β*3 (GSK3*β*) is ubiquitin-mediated and can degrade cytosolic *β*-catenin (*β*-catenin), thereby preventing *β*-catenin from accumulating in the nucleus. This prevents overexpression of downstream target genes, maintains the normal Wnt/*β*-catenin signaling pathway, and regulates cell division and migration [11, 21, 22]. APC mutation or deletion can lead to excessive activation of the Wnt/*β*-catenin signaling pathway, and the high expression of nuclear *β*-catenin can modify intercellular junctions to induce the progression of epithelial-mesenchymal transition (EMT). This eventually leads to abnormal cell proliferation, perturbed embryonic development, and tumorigenesis. Intestinal epithelial cell over-proliferation combined with insufficient apoptosis leads to the formation of intestinal adenoma nodules and colon adenocarcinoma, which ultimately contribute to invasive colon cancer [23]. APC mutations alter the balance between APC protein and *β*-catenin and E-cadherin, leading to changes in cell-cell and intercellular adhesion and contact inhibition. This disrupts the balance between cell division and cell death and becomes the rate-limiting factor of the proliferation process in the colorectum [24]. APC is an important tumor suppressor protein in the Wnt signaling pathway and mediates the development of colorectal cancer. The APC deletion leads to proliferation and volume increases of intestinal crypt cells, resulting in the formation of polyps.

FAP is an autosomal dominant genetic disease caused by mutations in APC [25]. As of April 2019, 1765 (2037) pathogenic APC mutations were recorded in the free version of the HGMD database. These include 421 missense/nonsense, 112 splice sites, eight regulatory sites, 721 small deletions, 310 small insertions, 43 small indels, 125 gross deletions, 12 gross insertions/duplications, 13 complex rearrangements, and repeat variations 0 pcs. A high frameshift mutation rate leads to enhanced APC inactivation. Among them, exon 16 represents 75% of the APC coding sequence and is also a hotspot mutation region [26]. In addition, 40–77% mutations are concentrated at the 5′ end of this region, which is a mutation-intensive region [27, 28]. Hutter et al. found the mutation of c.3260_3261del (p.Leu1087GlnQfs^*∗*^31) in a male 18-year-old proband of a FAP family for the first time, which is consistent with what we found [29]. The phenotypes of duodenal adenomas and colorectal adenomas are consistent with the family we found, but the difference is that there are two cases of bile duct polyp phenotype in the family we found. Earlier, we reported a rare case of bile duct polyp with special operation twice [8]. In the family we found, two cases with biliary polyps suggest that carriers of c.3260_3261del may be easily infected with biliary polyps. In addition, Jarry et al. found that there was a pathogenic mutation (c.3260_3263delTCAA) including the loss of two bases (c.3260_3261delTC) that we found in a FAP pedigree [30].

APC mutation sites are mostly located in codons 178–309 and 409–1580, whereas the most common pathogenic APC variant is located in codon 1309 (c.3927_3931delAAAGA). The general age of onset is 20 years old, which begins with a large number of colonic adenomas at the early stage. If not treated, the death of colorectal cancer patients with codon 1309 mutation, on average, occurs 10 years earlier than that of FAP patients carrying other mutations [31, 32]. The pathogenic variation in codons 1250–1464 can cause dense polyposis (average 5000 polyps) [33, 34]; however, this situation is not absolute [35]. AFAP (<100 colorectal adenomas) is associated with mutations in the mRNA alternative splice region before codon 157, after codon 1595, and exon 9. This is partly related to deletions within the APC. The APC pathogenic variant somatic mosaicism is usually associated with FAP. Extraintestinal manifestations of CHRPE pathogenic variants are related to codons 148–2043 or the deletion of the entire APC [35, 36]. APC mutation sites also affect colorectal pathological phenotypes. The missense mutation in codon 208 is related to the relatively mild colorectal pathological phenotype, the codon 367 mutation is related to AFAP attenuation, and the truncation mutation in codon 1309 can cause the highly malignant colorectal cancer pathological change phenotype. Mutations at codons 867 and 1114 and exons 6 and 9 affect the APC I*β*-catenin binding domain and are associated with a less severe colorectal cancer phenotype [37].

In addition to gastrointestinal endoscopic monitoring, regular inspection of other organs is particularly important because the disease spectrum due to APC mutation may involve disorders of multiple organs outside the intestine. For example, the incidence of desmoid tumors, osteoma, and epidermoid cysts is significantly higher in individuals carrying mutations in APC codons 1395–1493 than in individuals with mutations in codons 177–452, and the development of hepatoblastoma and/or brain tumors occurs when the pathogenic variant is located only in codons 457–1309 [25, 36, 38, 39]. Cancer in the mutation afflicted area is the main cause of death in FAP patients, and a biopsy should be performed in this area regardless of whether the mucosa is normal.

Since the occurrence and development of this disease is quite varied, significant differences are observed even among different individuals in the same family; therefore, the timing of treatment should be determined based on colonoscopy results of individual patients, rather than simply based on the gene mutation sites [40]. At present, preventive treatment remains the most important strategy for clinical management of patients with FAP. Monitoring via gastrointestinal endoscopy and weighing the risk of disease progression are the cornerstones of choosing endoscopic local treatment or preventive radical resection of the stomach and colon. Chemoprevention is defined as the use of drugs, natural medicines, or dietary supplements to reduce the incidence rate or delay the onset of disease (including cancer). Various chemoprevention strategies play key roles in delaying progression of polyps in patients with FAP, delayed prophylactic colectomy, and prevention of recurrence of adenomas after colectomy [16, 41, 42].

## Data Availability

The datasets used and/or analyzed during the present study are available from the corresponding author upon request.

## References

[1] Genetics Group of the Committee of Colorectal Cancer CA-cA (2018). Consensus on clinical diagnosis, treatment and pedigree management of hereditary colorectal cancer in China. *Zhonghua Zhong Liu Za Zhi*.

[2] Jo W.-S., Chung D. C. (2005). Genetics of hereditary colorectal cancer. *Seminars in Oncology*.

[3] Lockhartmummery P. (1925). Cancer and heredity. *The Lancet*.

[4] Alwahbi O. A., Abduljabbar A. S., Anwer L. A. (2018). Cancer in an unexpected site post pouch surgery for familial adenomatous polyposis (FAP). *International Journal of Surgery Case Reports*.

[5] Dinarvand P., Davaro E. P., Doan J. V. (2019). Familial adenomatous polyposis syndrome: an update and review of extraintestinal manifestations. *Archives of Pathology & Laboratory Medicine*.

[6] Ma H., Brosens L. A. A., Offerhaus G. J. A., Giardiello F. M., de Leng W. W. J., Montgomery E. A. (2018). Pathology and genetics of hereditary colorectal cancer. *Pathology*.

[7] Kobayashi H., Ishida H., Ueno H. (2017). Association between the age and the development of colorectal cancer in patients with familial adenomatous polyposis: a multi-institutional study. *Surgery Today*.

[8] Yan M.-L., Pan J. Y., Bai Y. N., Lai Z. D., Chen Z., Wang Y. D. (2015). Adenomas of the common bile duct in familial adenomatous polyposis. *World Journal of Gastroenterology*.

[9] Gao R., Liu Y., Gjesing A. (2014). Evaluation of a target region capture sequencing platform using monogenic diabetes as a study-model. *BMC Genetics*.

[10] Groden J., Thliveris A., Samowitz W. (1991). Identification and characterization of the familial adenomatous polyposis coli gene. *Cell*.

[11] Preisler L., Ben-Yosef D., Mayshar Y. (2019). Adenomatous polyposis coli as a major regulator of human embryonic stem cells self-renewal. *Stem Cells*.

[12] Horii A., Nakatsuru S., Ichii S., Nagase H., Nakamura Y. (1993). Multiple forms of the APC gene transcripts and their tissue-specific expression. *Human Molecular Genetics*.

[13] Anderson A., Plummer R., Abraham J. M. (2019). A unique cause of gastric polyposis. *Gastroenterology*.

[14] Munshi A. G., Hassan M. A. (2010). Common bile duct adenoma. *Surgical Laparoscopy, Endoscopy & Percutaneous Techniques*.

[15] Valerieva Y., Lutakov I., Golemanov B., Jelev G., Vladimirov B. (2018). A rare case of incidental common bile duct adenoma-endoscopic ultrasound evaluation. *Balkan Medical Journal*.

[16] Campos F. G., Sulbaran M., Safatle-Ribeiro A. V., Martinez C. A. R. (2015). Duodenal adenoma surveillance in patients with familial adenomatous polyposis. *World Journal of Gastrointestinal Endoscopy*.

[17] Offerhaus G. J. A., Giardiello F. M., Krush A. J. (1992). The risk of upper gastrointestinal cancer in familial adenomatous polyposis. *Gastroenterology*.

[18] Soravia C., Berk T., Madlensky L. (1998). Genotype-phenotype correlations in attenuated adenomatous polyposis coli. *The American Journal of Human Genetics*.

[19] Matsumoto T., Iida M., Kobori Y. (2002). Genetic predisposition to clinical manifestations in familial adenomatous polyposis with special reference to duodenal lesions. *The American Journal of Gastroenterology*.

[20] Björk J., Åkerbrant H., Iselius L. (2001). Periampullary adenomas and adenocarcinomas in familial adenomatous polyposis: cumulative risks and APC gene mutations. *Gastroenterology*.

[21] Liu P., Liang B., Liu M. (2020). Oncogenic mutations in armadillo repeats 5 and 6 of *β*-catenin reduce binding to APC, increasing signaling and transcription of target genes. *Gastroenterology*.

[22] Mariotti L., Pollock K., Guettler S. (2017). Regulation of Wnt/*β*-catenin signalling by tankyrase-dependent poly(ADP-ribosyl)ation and scaffolding. *British Journal of Pharmacology*.

[23] Kim W. K., Kwon Y., Jang M. (2019). *β*-catenin activation down-regulates cell-cell junction-related genes and induces epithelial-to-mesenchymal transition in colorectal cancers. *Scientific Reports*.

[24] Lugli A., Zlobec I., Minoo P. (2007). Prognostic significance of the wnt signalling pathway molecules APC, *β*-catenin and E-cadherin in colorectal cancer?a tissue microarray-based analysis. *Histopathology*.

[25] Plevová P. (2019). An update on inherited colon cancer and gastrointestinal polyposis. *Klinicka Onkologie*.

[26] Beroud C., Soussi T. (1996). APC gene: database of germline and somatic mutations in human tumors and cell lines. *Nucleic Acids Research*.

[27] Huang J., Zheng S. (2001). Characteristics of APC gene mutations in colorectal tumors. *Zhonghua Yi Xue Yi Chuan Xue Za Zhi*.

[28] Wu Z. G., Chen M. Q., Dong J., Peng Y., Mao J. F., Wang M. (2010). Pedigree analysis and study of germline mutations in the APC gene in a kindred with familial adenoma-tous polyposis. *International Journal of Genetics*.

[29] Hutter P., Rey-Berthod C., Chappuis P. O. (2001). Molecular and clinical characteristics in 32 families affected with familial adenomatous polyposis. *Human Mutation*.

[30] Jarry J., Brunet J.-S., Laframboise R. (2011). A survey of APC mutations in Quebec. *Familial Cancer*.

[31] Caspari R., Friedl W., Mandl M. (1994). Familial adenomatous polyposis: mutation at codon 1309 and early onset of colon cancer. *The Lancet*.

[32] Gayther S. A., Wells D., SenGupta S. B. (1994). Regionally clustered APC mutations are associated with a severe phenotype and occur at a high frequency in new mutation cases of adenomatous polyposis coli. *Human Molecular Genetics*.

[33] Nagase H., Miyoshi Y., Horii A. (1992). Correlation between the location of germ-line mutations in the APC gene and the number of colorectal polyps in familial adenomatous polyposis patients. *Cancer Research*.

[34] Newton K., Mallinson E., Bowen J. (2012). Genotype-phenotype correlation in colorectal polyposis. *Clinical Genetics*.

[35] Nieuwenhuis M. H., Vasen H. F. A. (2007). Correlations between mutation site in APC and phenotype of familial adenomatous polyposis (FAP): a review of the literature. *Critical Reviews in Oncology/Hematology*.

[36] Friedl W., Aretz S. (2005). Familial adenomatous polyposis: experience from a study of 1164 unrelated German polyposis patients. *Hereditary Cancer in Clinical Practice*.

[37] Ficari F., Cama A., Valanzano R. (2000). APC gene mutations and colorectal adenomatosis in familial adenomatous polyposis. *British Journal of Cancer*.

[38] Kashfi S. M., Behboudi Farahbakhsh F., Golmohammadi M., Nazemalhosseini Mojarad E., Azimzadeh P., Asadzadeh Aghdaie H. (2014). Frameshift mutations (deletion at codon 1309 and codon 849) in the APC gene in Iranian FAP patients: a case series and review of the literature. *International Journal of Molecular and Cellular Medicine*.

[39] Plawski A., Slomski R. (2008). APC gene mutations causing familial adenomatous polyposis in Polish patients. *Journal of Applied Genetics*.

[40] Friedl W., Caspari R., Sengteller M. (2001). Can APC mutation analysis contribute to therapeutic decisions in familial adenomatous polyposis? experience from 680 FAP families. *Gut*.

[41] Schönthal A. H., Chen T. C., Hofman F. M., Louie S. G., Petasis N. A. (2008). Celecoxib analogs that lack COX-2 inhibitory function: preclinical development of novel anticancer drugs. *Expert Opinion on Investigational Drugs*.

[42] Kim B., Giardiello F. M. (2011). Chemoprevention in familial adenomatous polyposis. *Best Practice & Research Clinical Gastroenterology*.

